# REPLACING THE CHAOS

**DOI:** 10.31486/toj.24.5042

**Published:** 2024

**Authors:** Ronald G. Amedee

**Affiliations:** Director of Clinical School and Professor, The University of Queensland Medical School, Ochsner Clinical School, New Orleans, LA; Editor-in-Chief, *Ochsner Journal*

*Goodness is stronger than evil; Love is stronger than hate; Light is stronger than darkness; Life is stronger than death….*

*–Desmond Tutu*

In 2020, 15 pregnancy-related deaths occurred in Louisiana, and 13 of those women were Black. The consequences are tragic, and the impact of each loss surges through families and communities. Even more tragic is the fact that the majority of pregnancy-related deaths are preventable. The Health, Medicine, and Society column in the Summer 2024 issue of *Ochsner Journal* goes beyond the statistics and tells the heart-wrenching story of a young mother who lost her life on postpartum day 7. In “The Fourth Trimester: Embracing the Chaos of the Postpartum Period,” Quebedeaux and Holman identify the barriers to care that underlie these preventable deaths and call on obstetrics providers to “embrace the chaos and work with patients to optimize care” during the critical postpartum period.

In addition to this important column, two original research articles, a literature review, a quality improvement article, and seven case reports and clinical observations make up the Summer issue.

The first original research article in this issue is authored by an Ochsner team that includes Ochsner Clinical School medical students, a senior statistician, and a senior physician. Feulner, Kossen, Lally, Ellis, Burton, and Galarneau contribute “Alcohol Misuse and Sexually Transmitted Infections: Using the CAGE Questionnaire as a Screening Tool,” a study of the correlations between a positive score on a common alcohol screening tool and the incidence of six sexually transmitted infections. According to the US Centers for Disease Control and Prevention (CDC), more than 2.5 million cases of syphilis, gonorrhea, and chlamydia were reported in the United States in 2022, making routine screening and early treatment a public health priority.

Investigators at the Charles E. Schmidt College of Medicine at Florida Atlantic University in Boca Raton, Florida, explored another topical public health issue by mining the CDC Youth Risk Behavior Survey for data regarding e-cigarette use by high schoolers. Hennekens, Adele, Mejia, Levine, and Kitsantas report their results in the second original research article, “Electronic Vapor Products: Alarming Trends in United States Adolescents.”

Most of the authors of the literature review in this edition are Ochsner Clinical School students with oversight provided by Dr David Galarneau. Dhillon, Feulner, Beitollahi, Kossen, and Galarneau address another contemporary issue in “At a Crossroads: Opioid Use Disorder, the X-Waiver, and the Road Ahead,” an eye-opening look at past and present opioid crises in the United States along with the concurrent US legislation that in some cases exacerbated these public health emergencies.

Studies have shown that reporting of medical errors, near misses, and adverse events is low among resident physicians even though the Accreditation Council for Graduate Medical Education requires that all residents participate in the reporting of patient safety events. Graduate medical education leaders Craig, Smith, and Shaeffer at UnityPoint Health in Des Moines, Iowa, developed a quality control project to increase reporting rates, and they share the results in “Improving Resident Physician Participation in Reporting Patient Safety and Quality Concerns.” A unique aspect of their project was the creation of a feedback mechanism to inform the reporting resident how the incident/problem was addressed.

Four of the seven case reports in this issue are by Ochsner staff. Neurology and Interventional Radiology contribute “Intrafacet Spacer Placement as a Mobility-Sparing Bailout Option in Atlantoaxial Fusion Construct Salvage,” authored by Scullen, Milburn, Mathkour, Tubbs, and Kalyvas. “Stumped by a Case of Appendicitis After Appendectomy” was authored by Ochsner Clinical School student Yuen and Emergency Medicine physician Suessman. Ochsner Orthopedics provides two case reports for the issue: “Vague Presentation of Cat Scratch Disease in a Child” by Crowley, Desai, and Waldron, and “Patellar Tendon Reconstruction Using Tibialis Posterior Allograft for Treatment of Patellar Tendon Rupture After Bone-Patellar Tendon-Bone Anterior Cruciate Ligament Reconstruction” by Renshaw, Few, Desai, Godshaw, and Jones.

As we begin the hot and humid southern summer, let us be ever mindful of the words of Desmond Tutu quoted above. In our current highly polarized society, Tutu's words are more meaningful than ever. While numerous important national and international issues and decisions lie ahead of us in the coming months, right now patients in our own communities face insurmountable challenges in accessing quality health care, safe and affordable housing, dependable transportation and childcare, first-rate education, living-wage employment, nutritious food, and the means to overcome language and literacy deficits. While these challenges are significant, Quebedeaux and Holman, in their column about the tragedy of maternal mortality, give us a way to approach these challenges in individual, human terms rather than as unsolvable, overwhelming problems: ***“Ultimately, by using humanistic approaches and acknowledging our patients’ struggles, we can replace chaos with education, empowerment, and healing.”*** Archbishop Tutu, who spent his life working to abolish apartheid and advance human rights, would have supported this approach.

